# Unique Features of Eye Movements Link to Cognitive and Mobility Decline

**DOI:** 10.1093/geroni/igaf122.2084

**Published:** 2025-12-31

**Authors:** Qu (Teresa) Tian, Yang An, Susan Resnick, Luigi Ferrucci

**Affiliations:** National Institute on Aging, Baltimore, Maryland, United States; National Institute on Aging, Baltimore, Maryland, United States; National Institute on Aging, Baltimore, Maryland, United States; NIA, Bethesda, Maryland, United States

## Abstract

Eye movement or ocular function, quantified by eye tracking devices, is a vital indicator of neurodegenerative diseases and behavior. However, existing knowledge is limited to patient samples or cross-sectional analysis. Little is known about eye movement associations with age-related cognitive and mobility decline. We investigated a diverse array of eye movement features in association with longitudinal changes in cognition and mobility in community-dwelling adults from the Baltimore Longitudinal Study of Aging (n = 543, mean age=71 years, 58% women, 25% Black, 5% cognitive impairment). Of 130 eye movement features derived from a portable eye-tracking device (Neurolign Dx100), 81 with <20% missing data were used. Of these 81, 40 features were significant predictors of age using LASSO regression and were used to create composite scores by category (saccade, smooth pursuit, vergence, optokinetic nystagmus). We examined the associations between eye movement composite scores and longitudinal changes in cognition, mobility, balance, manual dexterity, and gesture imitation up to 20 years using linear mixed-effects models, adjusted for age, sex, and race. Higher saccade and vergence scores were associated with less decline in executive function (Trail Making Test-part B), processing speed (Digit Symbol Substitution Test), mobility, balance, manual dexterity (Pegboard), and gesture imitation performance (all p < 0.05). A higher smooth-pursuit score was associated with less decline in mobility and balance. A higher OKN score was associated with less decline in balance. Selected eye movement features link to age-related cognitive and mobility decline in an aging population. Future studies are warranted to investigate underlying neuroimaging markers and brain pathology.

